# Evaluation of the Effects of Di-(2-ethylhexyl) phthalate (DEHP) on *Caenorhabditis elegans* Survival and Fertility

**DOI:** 10.1007/s12010-024-05032-z

**Published:** 2024-08-01

**Authors:** Alper Zöngür

**Affiliations:** Gemerek Vocational School, Sivas Cumhuriyet University, Sivas, Turkey

**Keywords:** Di-(2-ethylhexyl) phthalate (DEHP), *C. elegans*, Make it endocrine disrupter

## Abstract

Di-2-ethylhexyl (DEHP), which is widely used in industrial products, is produced annually in excess of 2 million tons worldwide. DEHP is an endocrine disruptor and one of the major environmental pollutant chemicals (EDCs) in nature. There is some information about the effects of these products, which provide great advantages in every respect, on human health and the environment. In this study, *C. elegans* organism was used to evaluate the health and environmental risks of DEHP. The survival and fertility effects of DEHP on the *C. elegans* organism were examined and the results were evaluated. In the study, it was determined that DEHP not only shortened the survival time of *C. elegans* but also caused a decrease in fertility. DEHP (0.625 mM and 10 mM) caused a 23.2–30.6% decrease in fertility. Additionally, the LC_50_ (50% lethal concentration) value of DEHP was found to be 321 µg/mL.

## Introduction

Phthalates are industrial compounds having a common chemical structure and dialkyl or alkyl/aryl esters of 1,2-benzenedicarboxylic acid. Phthalates are used for a variety of purposes, including personal care products (e.g. perfumes, lotions, cosmetics), paints, industrial plastics, some medical devices and medicines [15]. Some phthalates are commonly added to commercial products. In particular, phthalates are used to give flexibility to a rigid polyvinylchloride (PVC) [8]. Di-(2-ethylhexyl) phthalate (DEHP) is the most widely used phthalate ester and is produced worldwide over 2 million tons per year [26]. These widely used phthalates pollute food and the natural environment due to evaporation and leakage [5, 22]. In particular, foods consumed by humans are a source of exposure to DEHP. For example, there are many phthalate-containing products used for food packaging and processing, and these products are the source of the presence of DEHP in food [13, 34, 37]. Therefore, humans are exposed to phthalates, which are anthropogenic environmental pollutants, throughout their lives.

Phthalates can cause fetal death, testicular damage, liver damage, anti-androgenic activity, teratogenicity, peroxisome proliferation and especially reproductive toxicity in laboratory animals [36]. In particular, studies have shown that phthalates have hepato-carcinogenic, developmental and fertility effects. Additionally, studies have shown that phthalates have the potential to alter hormone action as they are endocrine disruptors or modulators. Therefore, these chemicals are thought to interfere with the function of the endocrine system, which is responsible for growth, sexual development, and many essential physiological functions in both male and female rats [17]. DEHP has a toxic effect on reproductive behavior by affecting endocrine system mechanisms in both male and female rats. In particular, studies have reported decreased testosterone levels and increased Leydig cell counts in rats after DEHP application [12, 18]. In addition, studies have shown that administration of DEHP to pregnant female rats can interfere with sexual differentiation by inducing a syndrome similar to testicular dysgenesis syndrome in male offspring. In conclusion, DEHP has been shown to target similar sites in the testis (Sertoli cells) and ovary (granulosa cells) [1, 32]. In a study, evaluated serum biomarkers such as estradiol, testosterone, anti-Müllerian hormone, tetraiodothyronine, thyroid-stimulating hormone, adiponectin, and leptin in mice of both sexes exposed to different doses of DEHP. The results indicated that DEHP had sex-specific effects in the reproductive system (male rats) and thyroid (female rats) in both sexes [40].

Toxicology studies in animals have raised concerns about the effects of human exposure to phthalates. Studies have reported that patients were exposed to the plasticizer di-(2-ethylhexyl) phthalate (DEHP) from polyvinyl chloride (PVC) tubes and bags during dialysis. Reports indicate that DEHP has a migration coefficient of 7.7 μg/ml/h from hemodialysis tubes to plasma [31]. As another major source of exposure to DEHP in the human population is food residues (excluding occupational exposures, medical exposures, and non-dietary intakes in children), DEHP has been studied in more detail than other phthalate esters [24]. The European Food Safety Authority (EFSA) has determined the tolerable daily intake (TDI) for DEHP as 0.5 mg/kg [3].

DEHP has been the subject of research on health problems in pregnant women, babies and children due to its use in many consumer products [6]. In one study, high levels of DEHP were detected in the urine of people using personal care products and it was shown that there is a relationship between care products and DEHP [10]. Endocrine disrupting chemicals (EDCs) such as phthalates are known to have an aggravating effect on the female reproductive system, causing reproductive disorders such as endometriosis, uterine fibroids, polycystic ovarian syndrome, premature ovarian failure, menstrual irregularity, menarche and infertility [14]. Studies have reported that many different phthalate metabolites (DEHP, dibutyl, monoethyl, monomethyl and monoisobutyl) are seen in the urine of babies exposed to baby lotion, powder or shampoo [11, 35]. Because these high molecular weight phthalates are not covalently bonded to plastics, they can leach from used products over time.

Much of our toxicity information in biology comes from research using in-vivo and in-vitro models. Each model selected in toxicity tests has strengths and limitations. Mammalian laboratory animals have similar developmental pathways as humans. This advantage makes laboratory mammals often preferred in toxicity tests. However, toxicity studies using mammalian models are expensive and time consuming [41]. Therefore, *Caenorhabditis elegans* organism is used as an alternative to mammalian laboratory animals in many toxicology studies [2]. *C. elegans* is a nematode about 1 mm in length and has a 4-stage life cycle (L1, L2, L3, L4). *C. elegans* is used in toxicological studies due to the traceability of biological processes, its close to 70% similarity with the human genome, and the similarity of mammalian biochemical pathways and biological systems [21].

The study was conducted to determine the effects of the toxic chemical DEHP on the life processes of the *C. elegans* organism. Additionally, the study aimed to evaluate the toxicity of DEHP in terms of time and dose. For this purpose, information about the toxicity and LC_50_ value of DEHP was provided thanks to the lifespan and fertility data obtained from the *C. elegans* organism.

## Material and Methods

### Preparation of *C. elegans* Nematode Growth Medium (NGM)

Wild-type (N2) *C. elegans* and *E. coli* (OP50) were obtained from the *Caenorhabditis* Genetics Center (Minneapolis, USA). 1 lt of NGM agar was prepared (17 g agar, 2.5 g peptone and 3 g NaCl) and 1 mL of 1 M CaCl_2_, 1 mL of 1 M MgSO4, 25 mL of 1 M KPO_4_ buffer (pH 6), 1 mL of cholesterol solution (5 mg/ mL) was added. After autoclaving, NGM medium was poured into petri dishes (6 cm). Afterwards, *E. coli* OP50 (500 µL) required for the feeding of *C. elegans* was added to the midpoint of the petri dishes.

### *C. elegans* Synchronization

The Petri dish was washed with dH_2_O to loosen the *C. elegans* eggs. The solution was transferred to a centrifuge tube and 0.5 mL of NaOH (1 g/5 ml) and 1 mL of sodium hypochlorite were added. The tube was centrifuged at 3400 rpm for 5 min to pellet the released eggs. The precipitate (pellet) was transferred to a petri dish (NGM) containing *E. coli* OP50 with a pasteur pipette. The temperature was set at 20 °C in all studies. In addition, 3 parallels were used in the study.

### Survival Assay

**Fig. 1:**
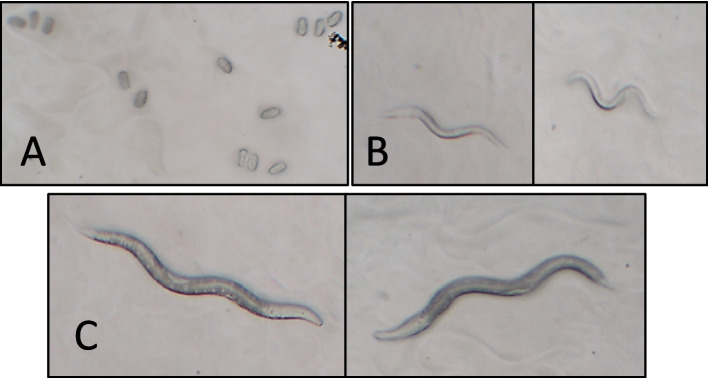
**A** Light microscope image (10x) of *C. elegans* eggs. **B** *C. elegans* L1 life form (4 × 10 stereo microscope). **C** *C. elegans* L4 life form (4 × 10 stereo microscope)

5-Fluoro-2′-deoxyuridine (250 µL, 50 mM FUDR), which inhibits *C. elegans* egg development, was added to NGMs used in survival analysis. Thus, the number of *C. elegans* in NGM was kept constant in the analyzes and fertility was prevented from affecting survival analyses. Synchronized 30 *C. elegans* (L4) were selected under a stereo microscope (Fig. 1C). Afterwards, 0.625 mM, 1.25 mM, 2.5 mM, 5 mM, 10 mM, concentrations were transferred to NGM plates prepared. Petri dishes containing DEHP and control were kept at 20 °C. Fresh *E. Coli* OP50 was added to NGM every 3 days. *C. elegans* individuals that died in the petri dishes at the same time every day were detected by stereo microscopy. Experiments were conducted until the last *C. elegans* died [39]. In the study, all tests were performed independently at 20 °C with 3 replications.

### Fertility Assay

Fifteen individuals from the synchronized L4 forms (*C. elegans* with developed gonads and hermaphroditic conical tips) (Fig. 1C) were taken under a stereo microscope and transferred to NGMs containing different concentrations of DEHP. Eggs seen in the medium after 36 h were counted with a light microscope (Fig. 1A). In the study, all tests were performed independently at 20 °C with 3 replications.

### Toxicity Analyzes (LC_50_)

DEHP concentrations of 0.01, 0.1, 1, 10, 20, 50, 100 µg/mL were used for LC_50_ tests. DEHP (0 mg/mL) was not added to the control groups. *C. elegans* L1 forms (Fig. 1B) were directly exposed to different concentrations (for 48 h) in eppendorf tubes [29]. The tests were repeated 3 times and their averages were used in statistical analysis.

### Statistics

Analyzes of the study were conducted with SPSS 26.00. Survival data were performed by Kaplan–Meier analysis. Additionally, Probit analysis was used to determine the amount of LC_50_. Averages were used in probit analyses. ANOVA-Post Hoc Test was used to determine whether there was a difference between the groups [30]. 

## Results and Discussion

### Survival Results

**Fig. 2:**
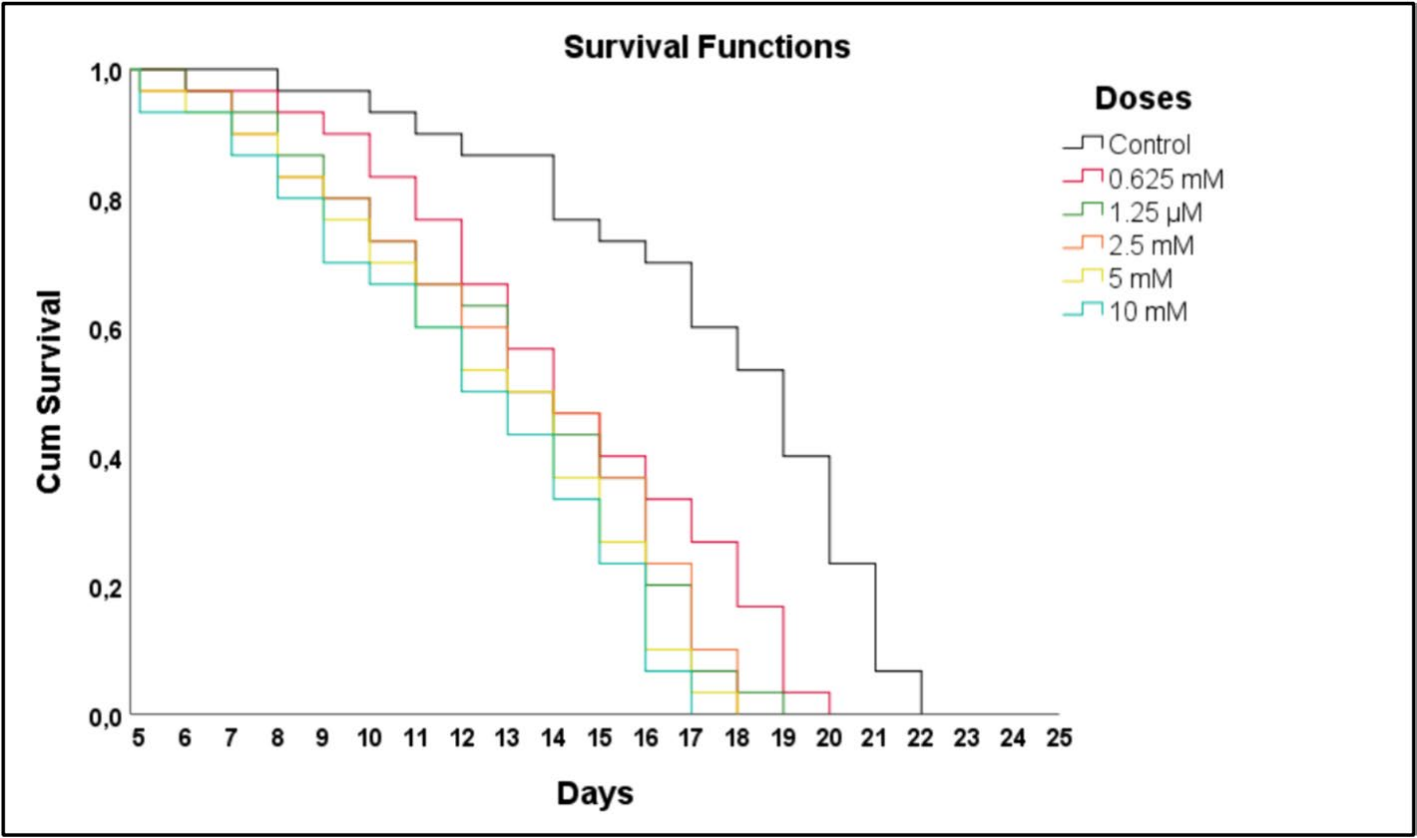
Kaplan–Meier analysis was performed according to different dose treatments and control. The survival function graph was created by averaging 3 replicate data

The *C. elegans* survival function of 5 different DEHP doses of treatment and control is shown in Fig. 2. At time zero (day one), the probability of survival is 100%. Although all *C. elegans* individuals in the doses administered on the first day and in the control group were alive, the number of deaths began to increase depending on the concentrations of the application doses from the 5th day. It was determined that *C. elegans* exposed to 10 mM, 5 mM, 2.5 mM, 1.25 mM, 0.625 mM DEHP doses died on the 17th, 18th, 18th, 19th, 20th day, respectively. Additionally, it was observed that all *C. elegans* in the control group died within 22 days. The mean and median survival days of *C. elegans* are shown in Table 1.

**Table 1:** The mean and median values of the data analyzed with SPSS 26.00 are shown

Means and Medians for Survival Time								
Doz	Mean^a^				Median			
	Estimate	Std. Error	95% Confidence Interval		Estimate	Std. Error	95% Confidence Interval	
			Lower Bound	Upper Bound			Lower Bound	Upper Bound
Kontrol	17,533	,391	16,768	18,299	19,000	,516	17,988	20,012
0.625 mM	14,267	,387	13,508	15,025	14,000	,631	12,763	15,237
1.25 mM	13,200	,374	12,468	13,932	13,000	,527	11,967	14,033
2.5 mM	13,133	,395	12,358	13,908	13,000	,678	11,672	14,328
5 mM	12,500	,374	11,767	13,233	13,000	,678	11,672	14,328
10 mM	12,067	,379	11,323	12,810	12,000	,632	10,760	13,240

a. Estimation is limited to the largest survival time if it is censored

### Fertility Results

The effect of different DEHP doses applied in the study on the number of *C. elegans* eggs is shown in Table 2. In the study, there were 739 eggs in the control group, 568 eggs in the 0.625 mM DEHP, 570 eggs in the 1.25 mM DEHP, 530 eggs in the in the 2.5 mM DEHP, 529 eggs in the 5 mM DEHP, and 513 eggs in the 10 mM DEHP. Additionally, the number of eggs counted in DEHP applications (0.625 mM, 1.25 mM, 2.5 mM, 5 mM, 10 mM) was compared with the control group (control group was considered 100%). The effectiveness was determined to be 69.4% at the highest dose (10 mM) and 76.8% at the lowest dose (0.625 mM). When the egg number averages were evaluated, it was seen that DEHP reduced *C. elegans* egg production by 23.2% to 30.6%. The effect of 5 different doses of DEHP on *C. elegans* egg rate was statistically significant. There was a significant difference between DEHP doses (p < 0.05).

**Table 2:** The number of eggs found with different dose treatments is shown

*C. elegans* egg numbers (fertility)						
repetition	Control	0.625 mM	1.25 mM	2.5 mM	5 mM	10 mM
1st	792	576	585	529	508	537
2nd	706	583	559	505	541	511
3rd	721	547	568	557	539	492
mean	739 ± 38	568 ± 16	570 ± 11	530 ± 21	529 ± 15	513 ± 18
% fertility	100 ^a^	76.8 ^ab^	77.1 ^ac^	71.6 ^a^	71.5 ^abc^	69.4 ^abc^

* According to ANOVA-Post Hoc test results, the average difference is significant at p < 0.05 level. The same letters indicate significant statistical differences between dose groups

### *C. elegans* LC_50_ Determination with Different DEHP Doses

**Fig. 3:**
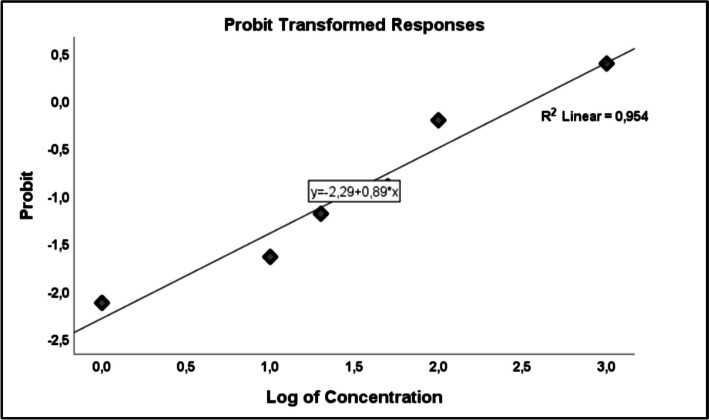
Probit analysis was performed with the survival data of *C. elegans* treated with different doses. Analyzes were repeated three times and averaged

*C. elegans* is an in vivo model used to study the impact and biochemical processes of toxicity [28]. In our study, the LC_50_ value of the DEHP molecule was determined as 321 µg/mL. This LC_50_ value was calculated by probit analysis based on the concentration at which 50% of *C. elegans* died (Fig. 3).

Since tests performed on *C. elegans* can provide rapid and consistent results, *C. elegans* can be used in ecological and health risk assessment tests of many toxicological chemical products. In our study, the effects of phthalate (DEHP) which is used especially in the production of children’s toys and plastic products on *C. elegans* lifespan and fertility were determined. In toxicity studies, it is important to reveal the statistical differences between dose increases of chemicals that affect *C. elegans* survival [45]. ANOVA analysis was performed using *C. elegans* survival analysis data and it was determined that there was a difference between the groups. DEHP (F(5, 534) = [26.738], p = [0.036]). Then, multiple comparisons were made with Post Hoc Test to determine between which dose groups these differences existed. When the results were evaluated, it was determined that there were significant differences between the control group and all dose groups (0.625 mM, 1.25 µM, 2.5 mM, 5 mM, 10 mM) (p < 0.05). While there was a significant difference between the 0.625 mM dose group and the 5 mM, 10 mM dose groups (p < 0.05), no significant differences were observed in multiple comparisons of other dose groups (1.25 µM, 2.5 mM, 5 mM, 10 mM) (p > 0.05). Statistical results showed that all doses of DEHP used in the study caused a shortening of the lifespan of *C. elegans*, but some dose increases did not cause significant differences. These results show that even a low dose of DEHP exposure has an impact on *C. elegans* survival. DEHP is one of the EDC. It is known that the incidence of mammary tumors increases and their latency period decreases after exposure to DEHP (150 mg/kg body weight/day) in female Sprague–Dawley (SD) rats [43].

**Fig. 4:**
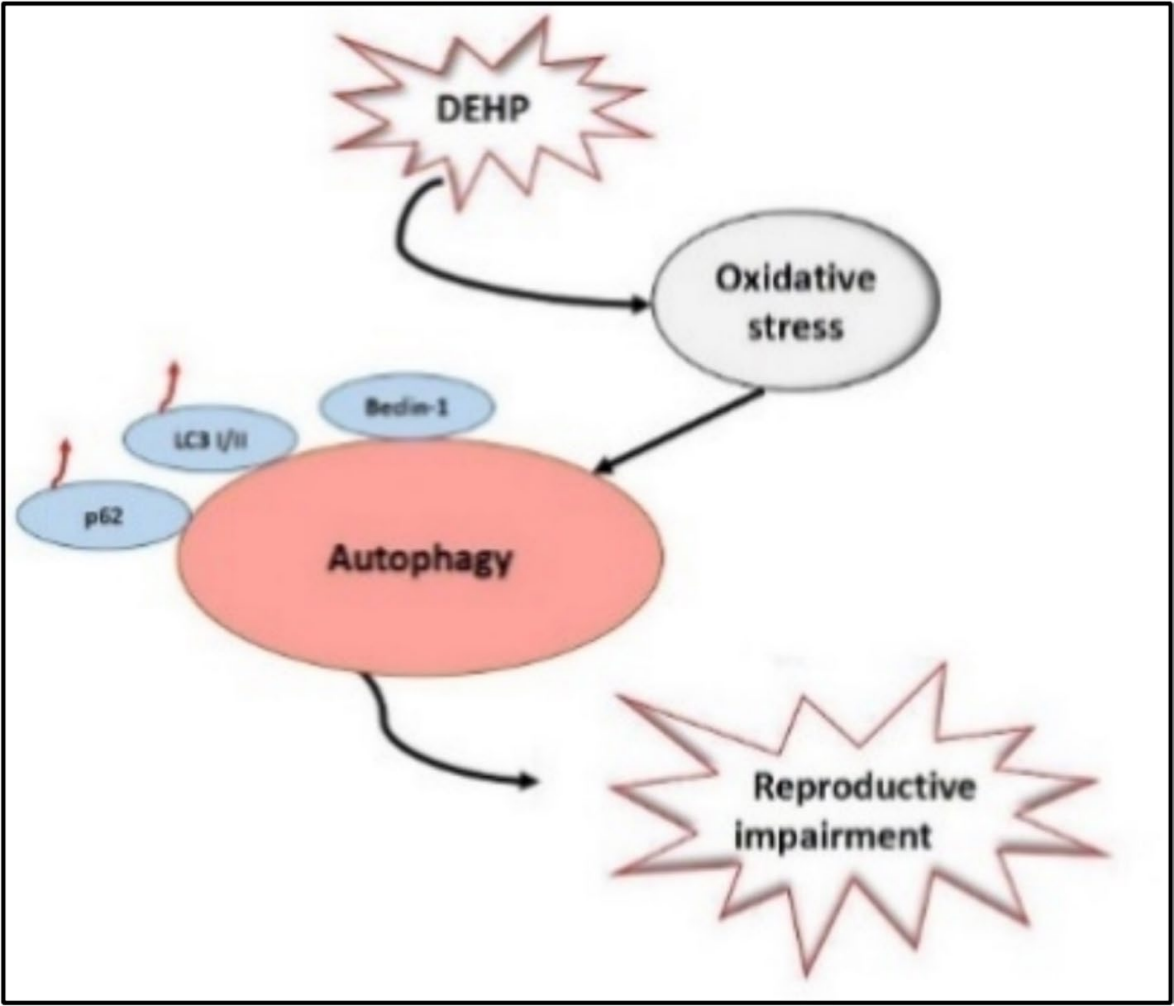
DEHP-induced ROS-mediated autophagy. DEHP increases reactive oxygen species (ROS). DEHP causes reduced growth of spermatogenic cells and infertility [20]

In the study, the effect of 5 different doses of DEHP on *C. elegans* egg rate was evaluated by ANOVA-Post Hoc test. A one-way ANOVA test revealed a statistically significant difference of DEHP on the fertility of *C. elegans*. DEHP (F(5, 576) = [81.124], p = [0.021]). In Post Hoc test, there were significant differences between control and all DEHP doses (p < 0.05). However, although there are numerical differences between the dose groups in Table 2 (except for the lowest and highest dose groups), no significant difference was seen between the dose groups in statistical tests (p > 0.05). This shows that all doses of DEHP used reduce egg numbers. It also explains that DEHP exposure causes a 25–30% decrease in egg production, regardless of the dose. Similarly, *C. elegans* germ cells exposed to DEHP have been reported to cause a decrease in egg laying rate and egg hatching ability [25]. *C. elegans* oocyte and distal tip cell (DTC) counts indicated that the number of oocytes decreased and apoptotic cells increased in groups exposed to 1 mg/L and 10 mg/L DEHP. Moreover, DEHP is known to affect the genes responsible for the estrogen biosynthesis process, causing a decrease in the number of eggs [27] (Fig. 4). Information on the LC50 value of DEHP on *C. elegans* is limited. In one study, the LC_50_ concentration after DEHP exposure has been reported to be over 100 mg/L [42]. Another recent study showed that exposure to DEHP (0 to 10,000 μg/L) caused reductions in offspring number, reproductive rate, reproductive length, and life expectancy [44]. Additionally, it has been reported that DEHP exposure causes a decrease in the gonad quantity of *C. elegans*. Although the DEHP LC_50_ values stated in the studies support our result, there is no complete numerical similarity. *C. elegans* is affected by environmental effects and the amount of nutrients in the NGM environment. In addition, especially the spawning status and life period (L1, L2, L3, L4, Dauer) of the *C. elegans* used in the studies affect the results of survival studies. Therefore, it is necessary to use FUDR in studies and exclude the fertility factor in studies, or to conduct studies paying attention to gender. In our study, it is thought that the determined LC_50_ value will make significant contributions to the literature since it was found by taking these situations into consideration. DEHP, which is used in many industrial products, has mutagenic effects that increase apoptotic and necrotic frequencies. It can also be said that DEHP has a toxic effect by triggering DNA damage in cells. In particular, one of the most important mechanisms that reduce lifespan and egg quantity in *C. elegans* is reactive oxygen species (ROS) [16, 23, 33]. Additionally, ROS accumulation is thought to bypass the defenses of *C. elegans* cells and trigger apoptotic mechanisms, leading to cell damage and ultimately death of *C. elegans* [20]. The defense mechanism against oxidative stress in *C. elegans* is the insulin/IGF-1 (IIS) signaling pathway. Binding of proteins enables DAF-2/IGFR activation. Thus, the cells themselves are protected. As oxidative stress increases, different serine/threonine kinases (AGE-1/PI3K, PDK-1, AKT-1/2, and SGK-1) phosphorylate DAF-16. Thus, phosphorylation and cytoplasmic sequestration are prevented [4]. Inhibition of the DAF-2 pathway leads to nuclear transport of DAF-16, HSF-1, and SKN-1, altering the expression profile of different genes involved in processes such as longevity, stress response, metabolism, or protein aggregation and refolding. Therefore, oxidative stress that impairs the function of ROS can damage numerous cellular processes. The fate of a cell with a damaged cell cycle checkpoint will be apoptosis. Increased levels of ROS occurring within mitochondria have the potential to induce downregulation of genes required for apoptosis by the early ROS-dependent signaling pathway [7]. There are a number of genes expressed in programmed cell death, including egl-1 and hus-1, which are clear markers of DNA damage-induced apoptosis [19]. Higher concentration (10 mg/L) of DEHP could inhibit the expression of autophagy genes (atg-18, atg-7, bec-1, lgg-1, and unc-51) [25]. Similarly, mice were administered DEHP at doses of 0, 125, 250 and 375 mg/kg/day. It has been reported that DEHP increased the liver function index of mice, lipid peroxidation, ROS, and expression of p53 protein [9]. Additionally, male mice were administered 0, 100, 200, or 400 mg DEHP/kg/day for 21 days. It has been stated that DEHP may cause the disintegration of the germinal epithelium and the decrease in sperm density in the epididymis, and that DEHP may induce the apoptosis of testicular tissue. The results showed that the decrease in serum testosterone concentration in the DEHP-treated group showed that DEHP could lead to Leydig cell damage. Furthermore, mouse TM3 Leydig cells were treated with 0–80 μM DEHP for 48 h and DEHP was noted to significantly inhibit cell viability and induce cell apoptosis [38].

In the study, it was determined that the survival time of the L1 forms (used in LC_50_ tests) of affected *C. elegans* was significantly reduced by changing the DEHP doses (p < 0.05). Additionally, the study determined that each change in DEHP dose (especially at higher doses) reduced the amount of eggs in the L4 form. Research data show the effect of DEHP on different life forms (L1, L4) of *C. elegans*. According to these results, it can be stated that direct exposure of babies, children and pregnant women to high doses of DEHP will cause serious health problems. Phthalates with high molecular weight can be found in plastic packaging products such as PVC products, toys and baby supplies, and cosmetic products. Because phthalates are not covalently bonded to plastics, they can leach from used products over time. This is one source of exposure to DEHP in products. Therefore, in many countries, the use of DEHP chemical should be banned, especially in baby products and children's toys.

Studies on *C. elegans* can be expanded to include mammalian laboratory animals. Thus, the effects of the dose amounts used in DEHP exposure on life expectancy and fertility can be explained more clearly. Additionally, the use of mammalian laboratory animals may be helpful in elucidating hormonal biochemical processes. However, the genome of *C. elegans* is similar to the human genome; It seems sufficient to emphasize the importance of the study data in terms of toxicity.

## Conclusion

Various micropollutants pose many risks when inhaled or absorbed through the skin. The presence of these chemicals in water and recycled water is a significant health risk. Micropollutants, especially those found in recycled water, raise concerns about possible human and ecological health problems. In this study, the effect of DEHP on human health was discussed using *C. elegans*. It appears that DEHP reduces both the lifespan and fertility of *C. elegans*. The results reveal the fact that DEHP is a significant threat to health and is considered an ecological risk. In summary, this study provides new insight into how chronic environmental exposure to DEHP may affect survival and fertility in *C. elegans* by affecting multiple molecular mechanisms.

## Data Availability

All data from this study are presented in the manuscript. It can be used if cited.
